# Preparation of Superhydrophilic/Underwater Superoleophobic and Superhydrophobic Stainless Steel Meshes Used for Oil/Water Separation

**DOI:** 10.3390/polym15143042

**Published:** 2023-07-14

**Authors:** Yu-Ping Zhang, Ya-Ning Wang, Hong-Li Du, Ling-Bo Qv, Jun Chen

**Affiliations:** 1 College of Chemistry and Materials Engineering, Hunan University of Arts and Science, Changde 415000, China; 2 College of Ecology and Environment, Zhengzhou University, Zhengzhou 450001, China; 3 College of Chemistry and Chemical Engineering, Henan Institute of Science and Technology, Xinxiang 453003, China

**Keywords:** stainless steel mesh, underwater superoleophobicity, chemical vapor deposition, superhydrophobicity, oil/water separation

## Abstract

Robust membrane materials with high efficiency have attracted extensive attention in oil/water separation. In this work, carbon particles via candle combustion were firstly adsorbed on the surface of stainless steel meshes (SSMs), which formed a thin hydrophobic coating, and a rough structure was then constructed through chemical vapor deposition and high temperature calcination, with the resultant SSM surface wrapped with uniform silica coating possessing the characteristic of superoleophobicity underwater. Scanning electron microscope (SEM), energy dispersive spectroscopy (EDS), and X-ray powder diffraction (XRD) were used to characterize the modified SSMs. The prepared SSMs were superhydrophilic in air, and they had superoleophobicity underwater (157.4°). The separation efficiency of five oil/water mixtures was above 98.8%, and the separation flux was 46,300 L·m^−2^·h^−1^. After it was immersed in 1 mol/L NaOH, 1 mol/L HCl and 3.5 wt% NaCl for 24 h, respectively, the efficiency was still above 97.3%. Further immersion in the solution of dopamine and octadecylamine resulted in the transformation of superhydrophililc/superoleophobicity-underwater SSMs to superhydrophobic SSMs, and the resultant SSMs with reverse surface wettability was also used for the oil/water separation with good separation efficiency and separation flux.

## 1. Introduction

The leakage of petrochemical products and the discharge of oily wastewater can cause serious ecological and environmental problems, so it is a serious challenge to select suitable methods and materials to treat oily wastewater quickly and efficiently [1,2,3]. At present, some traditional methods, such as adsorption [4], air flotation [5], flocculation [6], biological treatment [7], electrochemical treatment [8,9], and membrane filtration [10,11,12], are often applied for oil/water separation, but most of these separation methods have shortcomings, such as excessive energy consumption, low separation efficiency, long separation cycles, poor chemical and mechanical stability, and, sometimes, even secondary contamination [13,14].

With the rapid development of surface science and bio-nanotechnology, bioinspired materials with superwettability have been increasingly used in oil/water separation. The special properties of surface wettability mainly contain superoleophobicity, superhydrophilicity, superlipophilicity, and superhydrophobicity [15]. To achieve oil/water separation, it is necessary to have different wettability for water and oil, i.e., superhydrophobic/superoleophilic or super-hydrophilic/underwater superoleophobic. From the energy point of view, the surface tension of water is around 72.8 mN/m, while the surface tension of common organic phases is generally in the range of 20~40 mN/m. Therefore, materials that are hydrophilic in air must have a high surface energy, which determines that they are also necessarily lipophilic in air. However, if we want to use the hydrophilic material for oil/water separation, the special infiltration properties with superhydrophilicity in air and superoleophobicity underwater should be endowed [16].

Currently, oil/water separation is mainly achieved by super-hydrophobic/super-oleophilic materials, by which water is blocked but oil penetrates. In contrast, oil is blocked but water penetrates for superhydrophilic/underwater superoleophobic materials. Their separation properties are determined by the chemical composition of the surface and its microscopic geometry, and, often, the superwettability of the membrane material has to be modulated by constructing rough surfaces [17]. Superhydrophobic/superoleophilic materials have to treat the micro/nano-structures with low surface energy substances in order to repel water highly, and the hydrophilic chemical composition of the micro/nano-structures should be tailored in order to construct the superhydrophilic surfaces for the preparation of superhydrophilic/underwater superhydrophobic materials. 

Chen et al. prepared superhydrophobic SSM films by chemical etching and solution immersion. The separation efficiency of the mesh membrane modified with low surface energy substance of 1,2-hydroxystearic acid can be as high as 95.65% for petroleum ether and water, and the separation efficiency of the mesh membrane can still be maintained above 90% in weak acidic and alkaline as well as saline environments [18]. Ding et al. used the prepared hydrophobic and lipophilic SiO_2_ gels deposited on the surface of the SSM for the separation of kerosene and water, and the separation efficiency could still reach 97% after 40 cycles of oil/water separation [19]. Li et al. prepared a superhydrophobic SSM by spraying hydrophobic nano SiO_2_ on an SSM with carbon particles, which was adsorbed due to candle combustion. During the oil/water separation process, the surface nanoparticles were easily dislodged, and they easily lost their hydrophobic and lipophilic properties due to the poor attachment of the deposited carbon particles and hydrophobic nano-SiO_2_ to the smooth SSM [20]. Lei et al. used epoxy resin and fluorinated nano-graphite flakes to modify the surface of the SSM, and they prepared a superhydrophobic steel mesh with excellent separation of oil/water mixtures of *n*-hexane, *n*-decane, m-xylene, diesel, and methylene chloride, all with permeate fluxes above 41,000 L/(m^2^·h) and separation efficiencies up to 99.9% [21]. Inspired by the natural phenomenon that fish scales are not adhered by oil in water, Jiang et al. [22] coated a layer of hydrophilic polyacrylamide hydrogel (PAM) on SSM and obtained a surface with superhydrophilic-underwater superoleophobic properties, which successfully carried out the separation of oil/water mixtures. Cao et al. prepared superhydrophilic/underwater superhydrophobic membranes by in situ curing TiO_2_ nanoparticles and polyvinylpyrrolidone (PVP) onto SSM, and the contact angle of oil in water reached 160° and the oil/water separation efficiency was greater than 99.5%. The maximum membrane flux was 8422.5 L/(m^2^·h) [23]. Vollmer’s group used soot as a template to prepare robust transparent superamphiphobic glasses, and their work was published in the main issue of *Science* [24]. The authors first constructed a porous coating from candle ash on the glass slide and then modified the surface by chemical vapor deposition (CVD) [25,26], followed by calcination at 600 °C for about 2 h. After the entrapped carbon nanoparticles were removed, a robust superamphiphobic coating was obtained after further CVD with a low-surface energy substance. Referring to this method, Chao et al. prepared an identical glass flake and focused on investigating the anti-bacterial properties of the coating [27].

In this paper, a high strength, economical, and easily available SSM (SSM) is used as the substrate and a certain thickness of soot is adsorbed on the surface of the SSM through the candle combustion process, and silica is then uniformly deposited on the SSM surface by vacuum-assisted CVD of tetraethoxysilane (TEOS). The sol-gel method was catalyzed by ammonia solution [28,29], and the formed hybrid carbon/silica network was further calcinated, which led to the removal of carbon and intimate attachment of the silica network on the surface of SSMs. The resultant superhydrophilic/underwater superoleophobic SSMs were further modified to the superhydrophobic SSMs by the solution immersion of octadecylamine. Both SSWs with different surface wettability were successfully used for oil/water separation, and their separation performance and efficiency were investigated in detail.

## 2. Experimental Material and Method

### 2.1. Materials

Stainless steel mesh (304, 800 mesh) was purchased from Anping County Kai Zhong Wire Mesh Products Co., Ltd. (Anping County, Hengshui, Hebei, China). Tetraethyl silicate (GC, >99%), anhydrous ethanol (GC, ≥99.8%), carbon tetrachloride (AR), isooctane (AR), *n*-hexane (AR), *n*-octane (AR), petroleum ether (AR), dopamine, and octadecylamine (AR) were purchased from Shanghai Aladdin Biochemical Technology Co. Sodium hydroxide (AR, 96%), sodium chloride (AR, 99.5%), and ammonia (AR) were bought from Hunan Huihong Reagent Co. Candles were purchased from a local market. 

### 2.2. Preparation of Superhydrophilic/Underwater Superoleophobic (SiO_2_/SSM-1) and Superhydrophobic SSWs (SiO_2_/SSM-2)

**Figure 1:**
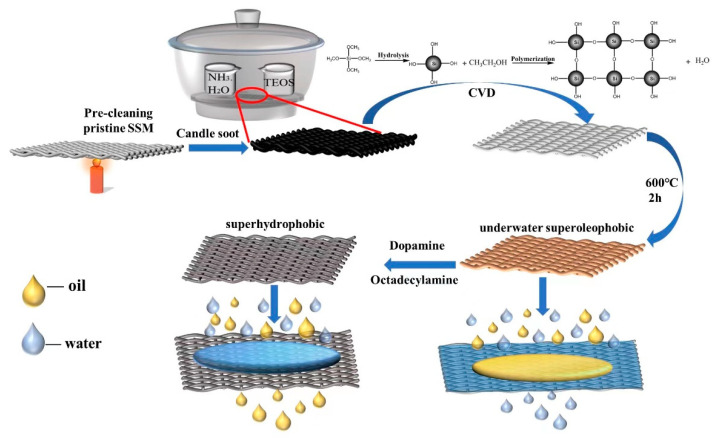
Preparation process of superhydrophilic/underwater superoleophobic SSMs and superhydrophobic SSMs.

The SSM was immersed in HCl (0.1 mol/L) and ethanol, deionized water, in turn, at room temperature, and ultrasonically cleaned for 30 min to remove oxidized material, oil, and other residual dirt. The preparation process of the SSMs and their application were illustrated in Figure 1. According to a previous report [30], the pretreated mesh surface was placed in a candle flame and the SSM was continuously moved back and forth and deposited for 3 min on each side, and then a black layer of candle soot was deposited on the surface of the mesh. Subsequently, it was placed in a vacuum desiccator with 1 mL of tetraethyl silicate (TEOS) and 1 mL of ammonia at the bottom of the desiccator, and then the desiccator was sealed and evacuated until the initial vacuum reached 0.2 MPa. The SSM was then deposited on both sides for 24 h. A layer of SiO_2_ was uniformly deposited on the soot layer through the cross-linking polymerization reaction of the TEOS. Finally, the above obtained SSM was placed in a muffle furnace and calcined at 600 °C for 2 h in order to remove the soot from the mesh, and the superhydrophilic/underwater superoleophobic SSM (denoted as SiO_2_/SSM-1) was obtained. The prepared SiO_2_/SSM-1 samples were then immersed in a mixed solution of 2 mg/mL dopamine hydrochloride, 10 mmol/L Tris, and 10 mmol/L octadecylamine. After this, they were stirred for 24 h at room temperature and the SSMs were taken out, washed with ethanol, and then dried in order to obtain the superhydrophobic SSMs (denoted as SiO_2_/SSM-2).

### 2.3. Instrumentation

The surface morphology of the SSM before and after modification was analyzed by SEM (ZEISS sigma500, Zeiss, Germany), the elemental distribution of SiO_2_/SSM was analyzed by EDS (BRUKER XFlash 6130, Oxford, UK), and the chemical composition of the SiO_2_/SSM surface was characterized by XPS (Thermo Fisher Nexsa, MA, USA). A contact angle analytical instrument (TST-300H, Shenzhen Tystein Co., Ltd., Shenzhen, China) was used to determine the surface wettability of the material. The SSM before and after modification was cut into 30 mm × 5 mm strips and fixed on the glass slides, and three samples in each group were then tested for water contact angle in air and oil contact angle under water, with the average value taken at least 3 times.

## 3. Results and Discussion

### 3.1. Microscopic Morphology and Composition Analysis

**Figure 2:**
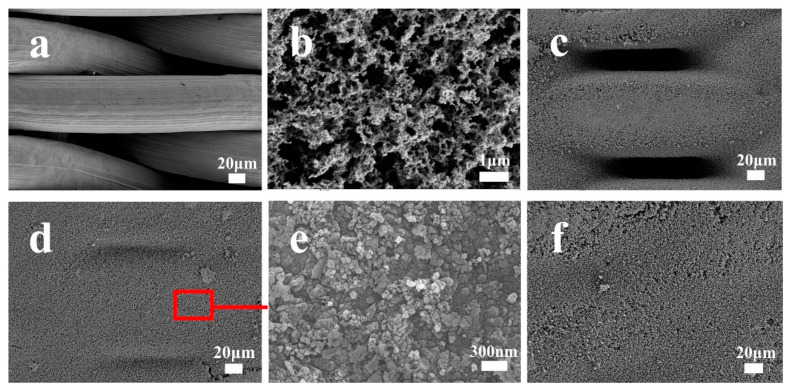
Pristine SSM (**a**); soot deposited layer (**b**); SSM after different CVD time of 16 h/24 h/36 h and calcination (**c**,**d**,**f**); silica coating at high magnification (**e**).

SEM was used to characterize the surface morphology of pristine SSM, SiO_2_/SSM-1. As shown in Figure 2a, the pristine SSM surface was smooth without obvious attached substances. Figure 2b showed the soot layer, and the metal mesh induced the incomplete combustion of the candle and produced a large number of soot particles, which uniformly covered the surface of the SSM. The SSM surfaces after CVD for 16 h, 24 h, and 36 h and after calcination are illustrated in Figure 2c,d,f, respectively. A small amount of nanoparticles were deposited on the SSM surface with a deposition time of 16 h, and the mesh aperture was still relatively obvious at this time. After 24 h deposition, the mesh size was obviously reduced, and the thickness of the silica nanoparticles covered on the surface was increasing. As the deposition time increased to 36 h, the presence of the mesh pores was almost invisible, and the thick deposition of SiO_2_ particles may have led to looser attachment on the SSM surface. Therefore, a deposition time of 24 h was chosen for the next application.

**Figure 3:**
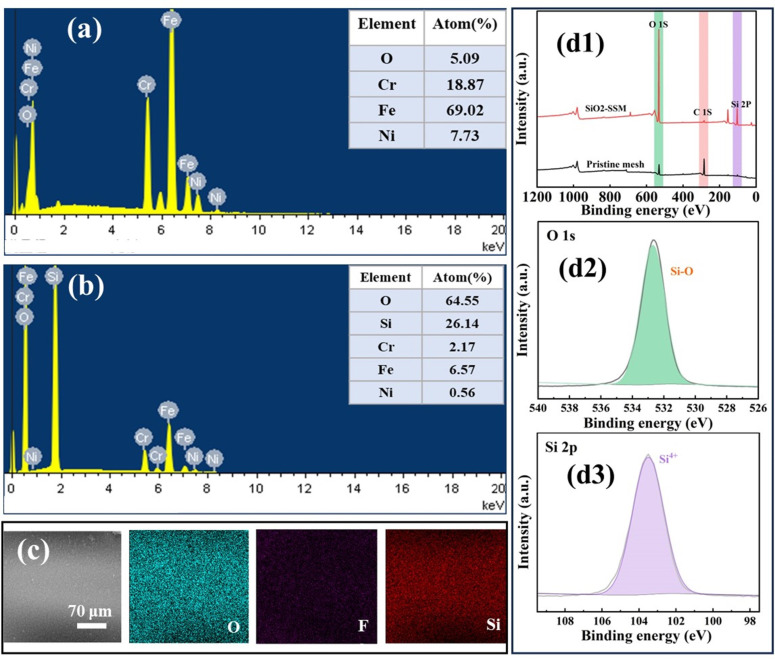
(**a**) EDS spectra of original SSM; (**b**) EDS spectra of SiO_2_ loaded SSM; (**c**) Element Mapping spectra of SiO_2_ loaded stainless steel mesh; (**d1**) XPS spectra of original SSM and SiO_2_ loaded SSM, (**d2**) O 1s peak fitting image, and (**d3**) Si 2P peak fitting image.

EDS results showed the elemental distribution of SSM before and after modification (see Figure 3a–c). Compared with the pristine SSM that O, Cr, Fe, and Ni were distributed uniformly on the SSM skeleton, with atomic ratios of 5.09%, 18.87%, 69.02%, and 7.73%, respectively, the comparative content of the four elements significantly changed, with atomic ratios of 64.55%, 2.17%, 6.57%, and 0.56%. The content of O was significantly increased, and, at the same time, the element Si appeared, which indicated that SiO_2_ is generated on the surface of the SSM.

The surface chemical bonds of the pristine SSM and SiO_2_/SSM-1 were further analyzed using XPS (Figure 3(d1–d3)). The positions of the characteristic peaks of the elements revealed the characteristic peak of O 1s at the binding energy of 532.65 eV, the characteristic peak of C 1s at the binding energy of 284.81 eV, and the characteristic peak of Si 2P at the binding energy of 103.51 eV. Compared with the pristine SSM, the SiO_2_/SSM has more intensive peaks of both O and Si elements, which proves the presence of SiO_2_. In the high-resolution XPS spectrum of O 1s (Figure 3(d2)), the peak fitted at 533.7 eV corresponded to the Si-O bond, and in the high-resolution XPS spectrum (Figure 3(d3)), the fitted peak corresponded to the Si^4+^ state, thereby confirming the generation of SiO_2_.

It is also difficult to observe the C peaks on the prepared SiO_2_/SSM-1, which indicates that the high temperature calcination removed the carbon particles thoroughly [23]. Herein, CVD led to the reaction of hydrolysis and condensation of TEOS, and the form of silica was similar to a Stöber reaction. Generally, the mass of silica layers deposited on the mesh in the absence of candle ash for about 24 h was calculated to be about 0.18 mg/cm^2^.

### 3.2. Wettability of the SSM Surface before and after Modification

**Figure 4:**
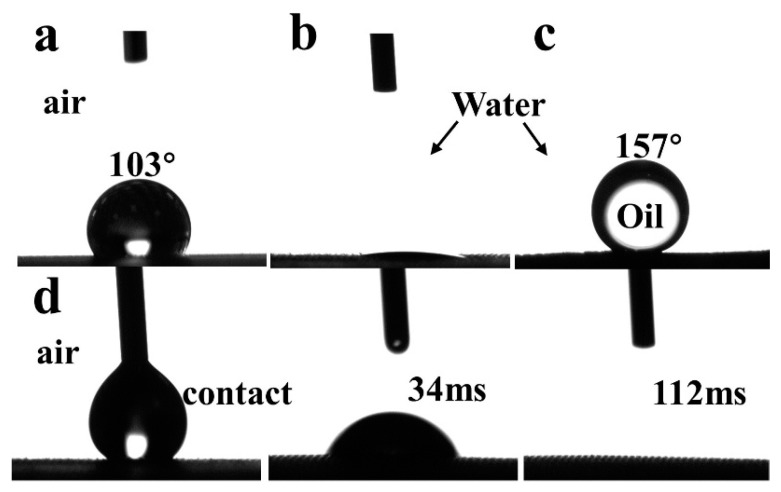
WCA on the pristine SSM in the air (**a**); UWOCA of octane on the pristine SSM (**b**); UWOCA of octane on the SiO_2_/SSM (**c**); and CA of octane on the SiO_2_/SSM in the air (**d**).

The wettability of the SSM before and after modification was analyzed using a contact angle tester. The water contact angle (WCA) and underwater oil contact angle (UWOCA) were both measured. As illustrated in Figure 4, the WCA in air on the pristine SSW was 103° ± 2.5° (Figure 4a) and the UWOCA of *n*-octane was close to 0° (Figure 4b), which indicated its typical superhydrophilic/underwater superoleophobic property. In air, the modified SSM was superhydrophilic, and when water droplets contacted the SiO_2_/SSM surface, they quickly spread on the mesh, reaching a CA of 0° in 112 ms (Figure 4d), whilst the average CA of *n*-octane underwater for three measurements was 157.4° ± 1.2° (Figure 4c).

This underwater superoleophobic structure is beneficial for oil/water separation [31,32]. In order to test the underwater superoleophobicity of different oils on the modified SSM, five organic solvents, namely, hexane, *n*-octane, isooctane, petroleum ether, and carbon tetrachloride, were selected for the test. As listed in Table 1, the density of carbon tetrachloride is 1.594 g/mL and can represent heavy oil and the other four organic solvents in the density between 0.65–0.703 g/mL can stand for light oil, and the surface tension of all five solvents is less than 30 mN/m. As illustrated in Table 1**,** the average values of UWOCA (*n* = 3) were 157.3°, 154.2°, 157°, 152.6°, and 148.5° for *n*-hexane, *n*-octane, *iso*-octane, petroleum ether, and carbon tetrachloride, respectively. This indicated that the modified SSM behaved obviously underwater in terms of superolephobicity and that it has potentially universal applicability for oil/water separation. Herein, it should be noted that the SSM without the soot layer did not exhibit underwater superoleophobicity in the presence of CVD for about 24 h.

### 3.3. Oil/Water Separation Performance for SiO_2_/SSM-1

**Figure 5:**
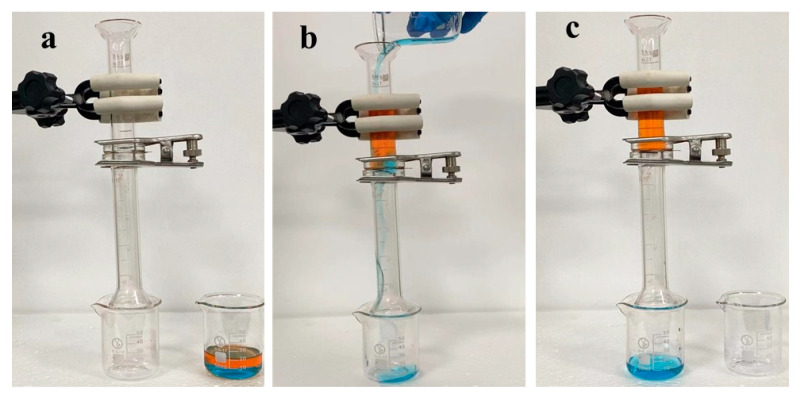
Oil (*n*-octane)/water separation process: (**a**) before separation; (**b**) in separation; (**c**) end of separation.

An immiscible solution of oil and water phases was poured onto the pre-wetted SiO_2_/SSM-1, which is fixed and sandwiched between two glass tubes. The oil/water separation process is shown in Figure 5. For the oil phase, *n*-hexane, *n*-octane, *iso*-octane, petroleum ether, and carbon tetrachloride was dyed red with Sudan III, respectively, and it was dyed blue with methylene blue for the water phase. The two phases were mixed according to V_oil_/V_water_ = 1/1, and were then poured into the glass tube from the top. When all of the deionized water entered the collection bottle below, timing was stopped and the spent time started being recorded. The whole separation process was carried out only under the action of gravity. Water penetrated quickly, and the oil was blocked above the SiO_2_/SSM-1. The separation mechanism can probably be attributed to the fact that the superhydrophilic surface of SiO_2_/SSM preferentially attracted the water phase in the three-phase system of oil/water/solid, and that the highly rough surface resulted in a quite small area fraction of the solid. Water molecules were tightly trapped in the rough SiO_2_/SSM-1 micro/nanostructures, which allowed the water phase to pass through the mesh by its gravity quickly, and the formed water barrier layer repelled the oil penetration. The separation efficiency was calculated by the following formula:

$$\eta =\frac{m_{1}}{m_{0}}\times 100\%$$

Here, m_0_ and m_1_ are the masses of water (g) before and after separation, respectively.

**Figure 6:**
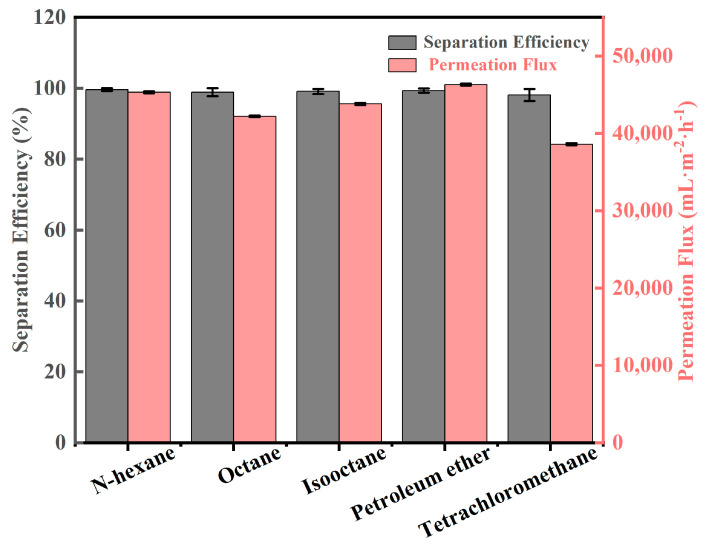
Separation efficiency and membrane flux of different oil/water mixtures by SiO_2_/SSM-1.

It can be seen that different mixtures of oil and water are separated efficiently with high separation fluxes using the SiO_2_/SSM [33]. Figure 6 shows the separation efficiencies and permeate fluxes of the different light oil and water mixtures. The separation flux was calculated by the following equation:

J = V/(S∙∆t)

where J(L·m^−2^·h^−1^) is the flux, V(L) is the filtrate volume, S(m^2^) is the effective area, and ∆t(h) is the permeation time.

The performance of oil/water separation was investigated with a separation efficiency higher than 98.1%, and permeation fluxes of water were in the range of 38,000 L·m^−2^·h^−1^~46,300 L·m^−2^·h^−1^. Moreover, after 20 cycles of separation, the separation efficiency was still greater than 97.3%, thus demonstrating its excellent stability and durability properties.

In the practical application of oil/water separation, the separation material usually needs to be used in harsh environments, so the chemical durability of the material is very important [34,35,36,37,38,39]. The fabricated SiO_2_/SSM-1 was thus immersed in 1 mol/L NaOH, 1 mol/L HCl, or 3.5 wt% NaCl (simulated seawater) solution, respectively. The UWOCA values of *n*-octane on the SiO_2_/SSM-1 were still 152°, 156°, and 154° with an oil/water separation efficiency greater than 95% after solution immersion for about 24 h. This demonstrates that the superhydrophilic underwater superoleophobic properties were not destroyed for our prepared robust SSMs.

### 3.4. Oil/Water Separation Performance for SiO_2_/SSM-2

**Figure 7:**
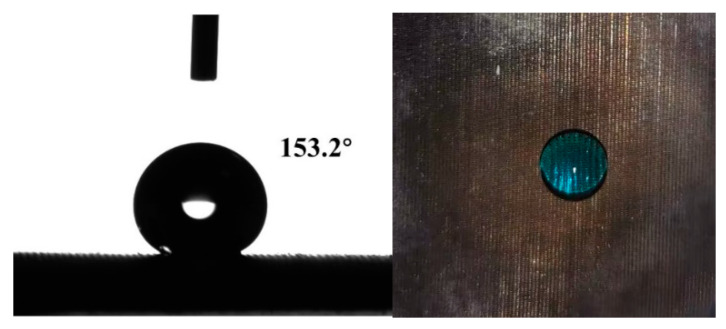
The images of superhydrophobic SSWs (SiO_2_/SSM-2).

After hydrophobic modification with a low-surface energy material of octadecylamine, the prepared SSMs with hierarchical structures possessed superhydrophobicity, which benefited the oil/water separation. As shown in the Figure 7, the water droplet (blue-dyed by methylthionine chloride) was seated spherically on the superhydrophobic surface of SSM-2, with a WCA of 153.2°.

**Figure 8:**
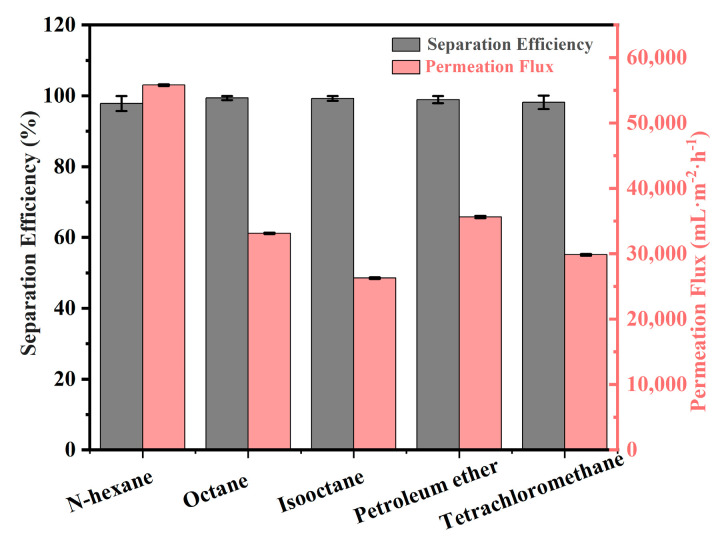
Separation efficiency and membrane flux of different oil/water mixtures by SiO_2_/SSM-2.

Additionally, the performance of oil/water separation for SiO_2_/SSM-2 was investigated in Figure 8, with a separation efficiency higher than 97.9 ± 2.1% and permeation fluxes of water in the range of 26,280 ± 117 L·m^−2^·h^−1^~55,800 ± 146 L·m^−2^·h^−1^. 

## 4. Conclusions

In this study, superoleophobic SiO_2_/SSM was prepared by sequentially depositing candle ash and silica nanoparticles on the surface of the SSM, followed by calcination at 600° for about 2 h. In addition, the SSM still maintained good superhydrophilic-underwater superoleophobic properties in harsh environments. Further chemical modification for the underwater superoleophobic SSMs created a superhydrophobic material. Both modified SSMs exhibited high-efficiency of oil/water separation, with a remarkable separation efficiency of about 98% or so, and a water permeate flux of more than 2.6 × 10^4^ L m^−2^ h^−1^. This work has developed a facile and versatile process for the fabrication of oil/water separation membranes without the use of environmentally harmful fluorinated substances.

## Figures and Tables

**Table 1:** Surface tension, density, and UWOCA of different liquids.

Liquid	Water	*n*-Hexane	*n*-Octane	Isooctane	Petroleum Ether	Carbon Tetrachloride
Surface Tension (m N/m)	72.8	20.3	22.6	20.5	16~22	26.77
Density (g/mL)	1	0.659	0.703	0.692	0.65	1.594
UWOCA (°)	0	163.3	158.2	160	164.6	148.5

## Data Availability

All data included in this study are available upon request by contacting the corresponding author.
